# Antifungal and Anti-Biofilm Effects of Caffeic Acid Phenethyl Ester on Different *Candida* Species

**DOI:** 10.3390/antibiotics10111359

**Published:** 2021-11-07

**Authors:** Ibrahim Alfarrayeh, Edit Pollák, Árpád Czéh, András Vida, Sourav Das, Gábor Papp

**Affiliations:** 1 Department of General and Environmental Microbiology, Faculty of Science, University of Pécs, Ifjúság Str. 6, 7624 Pécs, Hungary; pappgab@gamma.ttk.pte.hu; 2 Department of Biological Sciences, Faculty of Science, Mu’tah University, Mu’tah University Street, Al-Karak 61710, Jordan; 3 Department of Animal Anatomy and Developmental Biology, Faculty of Science, University of Pécs, Ifjúság Str. 6, 7624 Pécs, Hungary; peditmail@gmail.com; 4 Soft Flow Hungary R&D Ltd., Ürögi Fasor Str. 2/A, 7634 Pécs, Hungary; aczeh@foss.dk (Á.C.); avida@foss.dk (A.V.); 5 Department of Laboratory Medicine, Medical School, University of Pécs, Ifjúság Str. 13, 7624 Pécs, Hungary; pharma.souravdas@gmail.com

**Keywords:** CAPE, *Candida*, antifungal, biofilm, apoptosis

## Abstract

This study investigated the effect of CAPE on planktonic growth, biofilm-forming abilities, mature biofilms, and cell death of *C. albicans*, *C. tropicalis*, *C. glabrata*, and *C. parapsilosis* strains. Our results showed a strain- and dose-dependent effect of CAPE on *Candida*, and the MIC values were between 12.5 and 100 µg/mL. Similarly, the MBIC values of CAPE ranging between 50 and 100 µg/mL highlighted the inhibition of the biofilm-forming abilities in a dose-dependent manner, as well. However, CAPE showed a weak to moderate biofilm eradication ability (19-49%) on different *Candida* strains mature biofilms. Both caspase-dependent and caspase-independent apoptosis after CAPE treatment were observed in certain tested *Candida* strains. Our study has displayed typical apoptotic hallmarks of CAPE-induced chromatin margination, nuclear blebs, nuclear condensation, plasma membrane detachment, enlarged lysosomes, cytoplasm fragmentation, cell wall distortion, whole-cell shrinkage, and necrosis. In conclusion, CAPE has a concentration and strain-dependent inhibitory activity on viability, biofilm formation ability, and cell death response in the different *Candida* species.

## 1. Introduction

The genus *Candida* refers to a yeast that is part of the microbiota of healthy individuals living in commensalism with the human. However, in some cases, few *Candida* species tend to be opportunistic fungal pathogens, causing candidiasis. Candidiasis is among the common human infections; its symptoms vary according to the location of the infection in the body. Most of the infections may lead to minor symptoms such as slight localized rashes, redness, itching, and discomfort, though symptoms can be severe or even fatal if left without treatment in immunocompromised individuals [1,2,3]. Mostly, candidiasis is attributed to *Candida albicans*, however; non-albicans *Candida* species, including *C. parapsilosis*, *C. tropicalis*, and *C. glabrata*, have been reported to cause 30% to 54% of *Candida* infections [4,5,6]. Furthermore, the ability of these species to exhibit multidrug resistance, which may cause failure of the antifungal therapy, has been reported in earlier studies [6].

The ability of *Candida* spp to shift the commensal to pathogenic lifestyle is attributed to the presence of several virulence factors. Predominantly, the capability of switching morphology between yeast and hyphal forms, and the ability to form biofilms are the major properties crucial to *Candida* spp pathogenesis. The *Candida* infections are accompanied by the formation of biofilms on host tissues, organs, or abiotic surfaces such as urinary catheters, which result in high morbidity and mortality [7,8,9]. As with all microbial biofilms, *Candida* biofilms are highly resistant to antimicrobial treatment. Several factors might contribute to this resistance, including the physiological state of *Candida* cells in the biofilm, extracellular polymeric substances, overexpression of membrane-localized drug efflux pumps, variations in sterol content in the fungal membrane, and different developmental phases of cells through the biofilm [10]. Thus, the effectiveness of the current therapeutic agents against *Candida* biofilms is considered low, with a few exceptions [7,8,9]. The biofilms of *Candida* were 30 to 2000 times more resistant than planktonic cells to many antifungal agents, including amphotericin B, fluconazole, itraconazole, and ketoconazole [11]. As a result, the need to provide natural alternatives to these synthetic antifungal agents has arisen. The antifungal mechanisms of action of these natural alternatives can include the inhibition of germination and biofilm formation, the disruption of cell wall integrity, the alteration of cell membrane permeability, or the induction of apoptosis [9].

Caffeic acid phenethyl ester (CAPE), also called phenylethyl caffeate or phenethyl caffeate, is one of the promising natural alternatives of synthetic antimicrobial drugs. This polyphenolic ester compound is a major component of temperate propolis (poplar-type) and can be produced in the laboratory by reacting caffeic acid with phenethyl alcohols. It consists of hydroxyl groups within the catechol ring, which is crucial for many biological activities [12,13]. No information was available in the literature about the LD_50_ of CAPE in animal models or on normal human cells. However, Koru and coworkers investigated cytotoxicity in the human multiple myeloma cell line with LD_50_ at 24, 48, and 72 h, found at 49.1, 30.6, and 22.5 μg/mL, respectively [14]. The CAPE has a wide range of biological activities, such as inhibition of nuclear factor κ-B, cell division restriction, termination of the cell cycle, and apoptotic induction [15]. It has been studied extensively as the most important individual component of propolis. Existing studies focused on its potential therapeutic properties such as its antibiotic, antioxidant, anti-inflammatory, anti-oxidative stress, antitumor, antidiabetic, anti-neurodegeneration, and anti-anxiety properties [13,16,17]. Numerous studies demonstrated the antibacterial activity of CAPE against different bacterial species [18,19,20,21,22]. However, few studies that have investigated the antifungal activity of CAPE as a single molecule or in combination with some antibiotics were found in the literature. De Barros and coworkers recently reported the ability of CAPE to inhibit the growth of both fluconazole-sensitive and fluconazole-resistant strains of *C. albicans* [23]. Moreover, Sun and coworkers found the synergistic effects of CAPE with caspofungin and fluconazole against *C. albicans* and fluconazole-resistant *C. albicans*, respectively [24,25]. The synergism with caspofungin was associated with a loss in iron homeostasis induced by CAPE, leading to functional defects in the mitochondrial respiratory chain and energy depletion, which increases the susceptibility of *C. albicans* to caspofungin [25].

Presently, CAPE has been given close attention for its important therapeutic effects in many diseases, including carcinomas, internal organ damage, metabolic diseases, inflammatory diseases, and microbial infections. CAPE may be a promising natural product for clinical application in the future [21]. One of the major advantages is that CAPE is devoid of some negative aspects of crude extracts of propolis, which includes, the inability to be standardized, which is a keystone of implementing it as therapy in the field of medicine [16]. This study aimed to investigate how CAPE, as a single molecule, affects planktonic growth, biofilm-forming abilities, mature biofilms, and cell death of some *Candida albicans* and non-albicans *Candida* species and strains.

## 2. Results

### 2.1. Susceptibility of Candida Planktonic Cells to CAPE

The antifungal effect of CAPE on nine *Candida* strains has been studied. The results of the minimal inhibitory concentration (MIC_80_) values for CAPE against different *Candida* species and strains are given in Table 1. It has been found that CAPE has a strain- and dose-dependent effect. The MIC_80_ values ranged from 12.5 to 100 µg/mL. The highest inhibitory effect was seen against *C. glabrata* SZMC 1378, *C. glabrata* SZMC 1374, and *C. parapsilosis* SZMC 8008 compared with the other strains. However, the most resistant strain was *C. albicans* SZMC 1423.

### 2.2. Effect of CAPE on Candida Biofilm-Forming Ability

**Figure 1:**
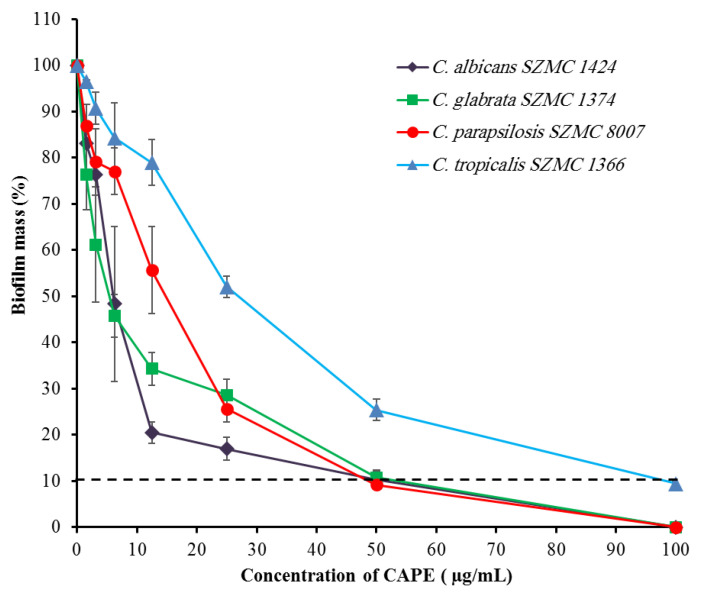
Effect of CAPE on the biofilm-forming ability of four *Candida* species. The dashed line represents the MBIC. Data are shown as mean ± SD from three independent experiments.

The biofilm-forming ability is a crucial property related to the pathogenicity of *Candida*. In this experiment, the effect of CAPE was tested on the biofilms of four different *Candida* strains with high biofilm-forming abilities. The results demonstrate that CAPE has a dose-dependent inhibitory effect on the biofilm formation in four strains (Figure 1). The minimal biofilm inhibitory concentration (MBIC) values were 50, 50, 50, and 100 µg/mL for *C. albicans* SZMC 1424, *C. glabrata* SZMC 1374, *C. parapsilosis* SZMC 8007, and *C. tropicalis* SZMC 1366, respectively.

### 2.3. Effect of CAPE on Candida Biofilm Eradication

**Figure 2:**
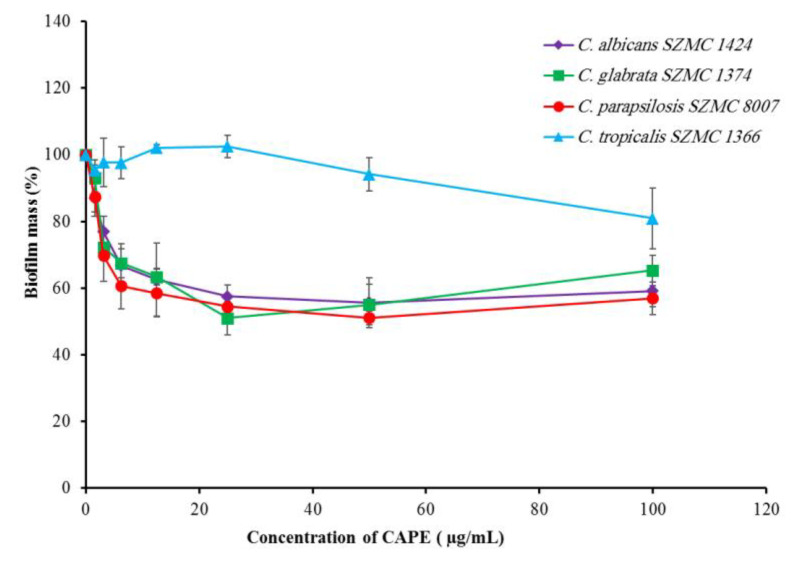
Effect of CAPE on mature biofilms of four *Candida* species. Data are shown as mean ± SD from three independent experiments.

The effect of CAPE on the mature biofilms of four biofilm-forming *Candida* species was investigated. Treatment of the mature biofilms of *C. albicans* SZMC 1424, *C. glabrata* SZMC 1374, *C. tropicalis* SZMC 1366, and *C. parapsilosis* SZMC 8007 with different concentrations of CAPE caused a partial eradication. The maximum eradications (19–49%) for the mature biofilms of *C. glabrata* SZMC 1374, *C. albicans* SZMC 1424, and *C. parapsilosis* SZMC 8007 were achieved at 25, 50, and 50 µg/mL, respectively. Moreover, the eradication process was found to be dose-independent above 50 µg/mL in the case of these strains. On the other hand, the mature biofilms of *C. tropicalis* SZMC 1366 were the most resistant to CAPE, and the maximum eradication was achieved at 100 µg/mL (Figure 2).

### 2.4. Biosorption of CAPE by Candida Cells

**Figure 3:**
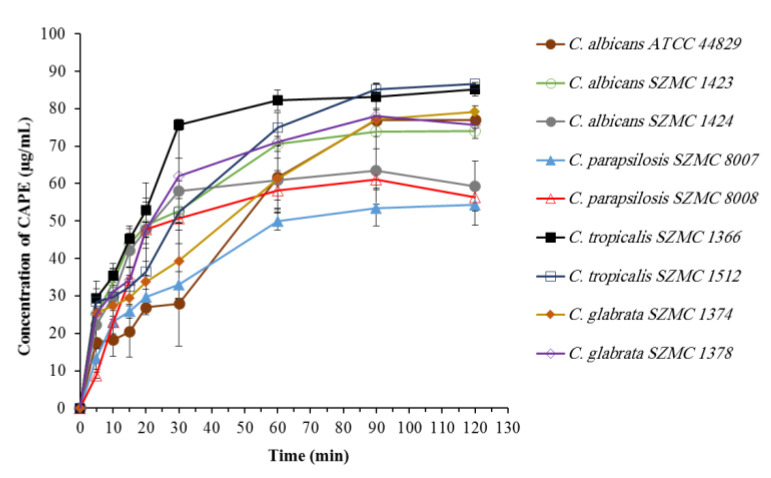
Cellular uptake of CAPE by nine *Candida* strains. Data are shown as mean ± SD from three independent experiments.

Biosorption may be defined as “the removal/binding of desired substances from aqueous solution by biological material” [26]. The results revealed that the biosorption of CAPE by different *Candida* strains occurs rapidly, followed by a maximum biosorption observed within the first 30 to 90 min (Figure 3). According to the amount of CAPE biosorbed, two groups can be recognized: the first group was able to biosorb 53–63 µg/mL of CAPE, and it includes *C. albicans* SZMC 1424, *C. parapsilosis* SZMC 8007, and *C. parapsilosis* SZMC 8008; in contrast, the second group was able to biosorb 74–86 µg/mL, and it includes *C. albicans* ATCC 44829, *C. albicans* SZMC 1423, *C. tropicalis* SZMC 1366, *C. tropicalis* SZMC 1512, *C. glabrata* SZMC 1374, and *C. glabrata* SZMC 1378.

### 2.5. Induction of Apoptotic Cell Death in Candida spp. by CAPE

**Figure 4:**
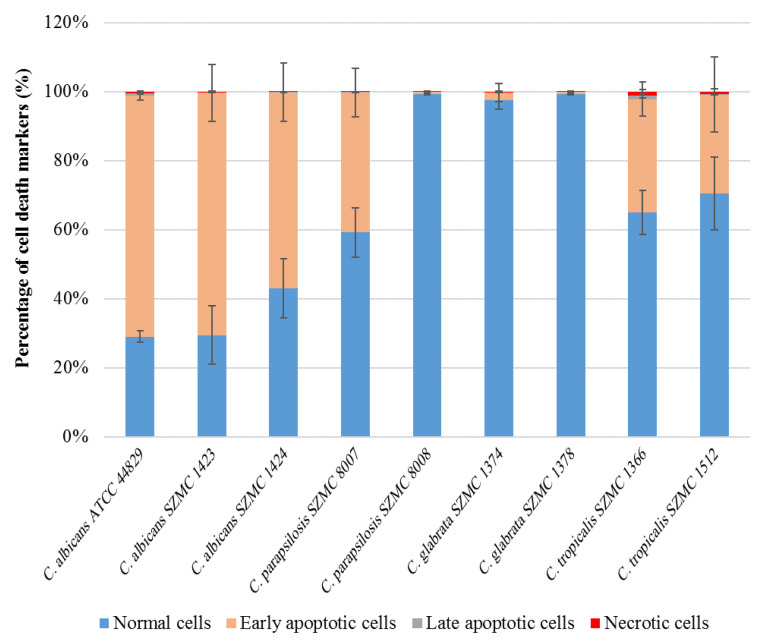
Cell death induced by CAPE treatment in nine *Candida* strains as determined by annexin V and PI staining. Data are shown as mean ± SD from three independent experiments.

Cells of the nine *Candida* strains treated with sub-lethal concentrations of CAPE were analyzed by double staining with CF^®^488A Annexin V and propidium iodide (PI). The apoptotic cells with externalized phosphatidylserine were detected by CF^®^488A Annexin V, while necrotic cells were detected by PI staining. The results shown in Figure 4 demonstrate CAPE-induced apoptosis in six of the tested strains at different levels. Among these strains, *C. albicans* ATCC 44829 and *C. albicans* SZMC 1423 revealed the highest percentage of early apoptotic cells (69.8 and 70.2%, respectively), whereas almost no apoptosis was seen in *C. glabrata* SZMC 1374, *C. parapsilosis* SZMC 8008, and *C. glabrata* SZMC 1378 (apoptotic cells ≤ 2%). On the other hand, no necrosis was observed in any of the tested strains (necrotic cells ≤ 1%). Examples of the scatter plots can be found in the supplementary material (Figures S1–S7).

### 2.6. Effect of Caspase Inhibitor on the Growth of CAPE-Treated Candida Cells

**Figure 5:**
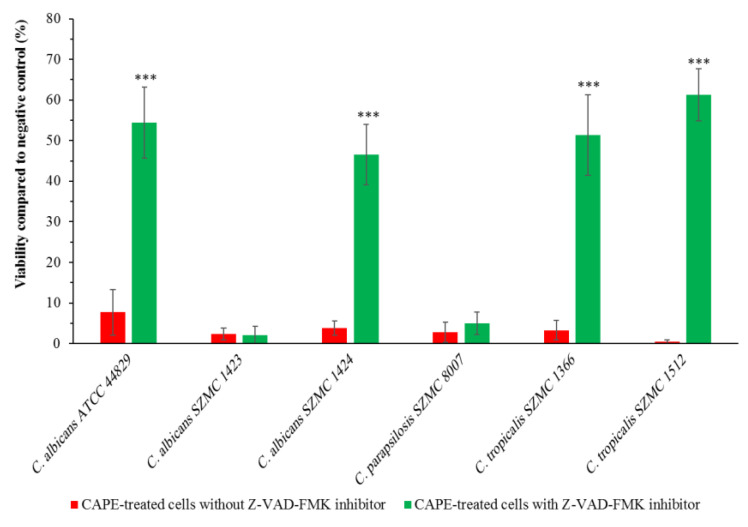
Effect of the pan-caspase inhibitor Z-VAD-FMK on the viability of six *Candida* strains treated with sub-lethal concentrations of CAPE. Data are shown as mean ± SD from three independent experiments. *** *p* <0.001 indicates a significant increment of the viability compared with the viability without pre-incubation with the pan-caspase inhibitor Z-VAD-FMK.

To investigate whether yeast caspase Yca1p is involved in CAPE-induced apoptotic cell death, pre-incubation with the pan-caspase inhibitor Z-VAD-FMK was applied for 1 h. The growth of the sub-lethal CAPE concentration-treated *Candida* strains that had apoptosis was analyzed with and without pre-incubation with Z-VAD-FMK. As shown in Figure 5, a significant increase in the viability was observed in *C. albicans* ATCC 44829, *C. albicans* SZMC 1424, *C. tropicalis* SZMC 1366, and *C. tropicalis* SZMC 1512 that are pre-incubated with the pan-caspase inhibitor Z-VAD-FMK. However, the viability of CAPE-treated *C. albicans* SZMC 1423 and *C. parapsilosis* SZMC 8007 was not affected by the pre-incubation with the pan-caspase inhibitor Z-VAD-FMK.

### 2.7. CAPE-Induced Subcellular Cell Death Markers Determined by TEM

**Figure 6:**
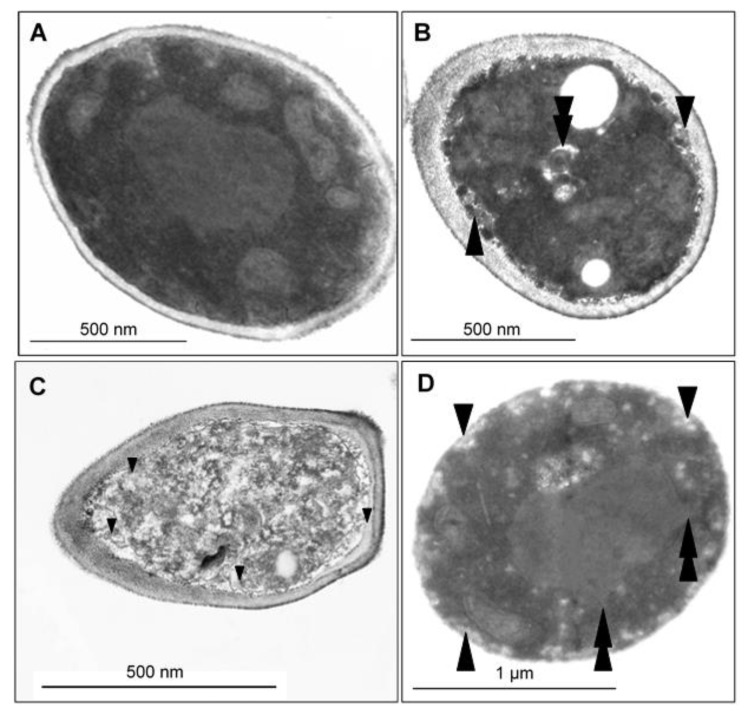
(**A**) TEM micrographs of the control *Candida tropicalis* SZMC 1366 cell structure demonstrates intact membranes, small unevenly scattered condensed chromatin grains, and homogenous cytoplasm structure. (**B**–**D**) TEM micrographs of *C. tropicalis* treated with a sub-lethal concentration of CAPE: (**B**) Late-stage disintegration with membrane fingerprints, vacuolization, plasma membrane detachment (arrowheads), and vacuole formation (double arrowheads). Fine granular homogenous cytoplasm organization disappeared, and a dense, compact cytoplasm with signs of fragmentation, rounded cell shape, and whole-cell shrinkage was seen. (**C**) Necrotic cell with membrane ruptures (arrowheads) and loss of cytoplasm density. (**D**) Several peripheral vacuoles show plasma membrane involvement (arrowheads). Nuclear bleb formation (double arrowheads).

**Figure 7:**
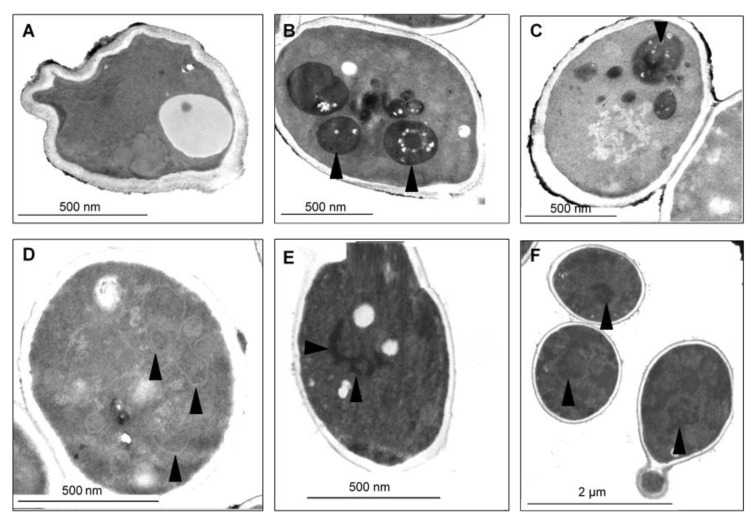
TEM micrographs of *C. albicans* SZMC 1423 treated with a sub-lethal concentration of CAPE exhibit different markers of cellular deterioration. (**A**) Severe cell wall distortion. (**B**,**C**) Appearance of enlarged lysosomes was rather frequently detected (arrowheads). (**D**) Isolation membranes precondition of cytoplasm fragmentation (arrowheads). (**E**,**F**) Nucleus fragmentation and marginal condensation (arrowheads).

**Figure 8:**
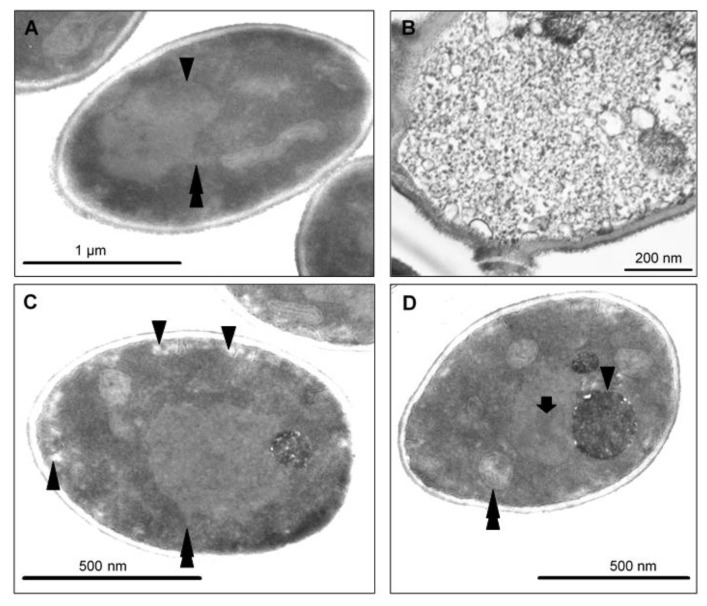
TEM micrographs of *C. parapsilosis* SZMC 8007 treated with a sub-lethal concentration of CAPE. Both apoptotic and necrotic cell structural changes were observed in samples. (**A**) Nuclear chromatin margination and condensation (arrowhead) and blebs (double arrowhead) detached from the nucleus are typical apoptotic hallmarks. (**B**) Few necrotic cells were also present. Note membrane disintegration, obvious vacuolization, and loss of cytoplasm density. (**C**) Peripheral vacuole formation refers to Golgi fragmentation and cell membrane separation from the cell wall (arrowheads). Nuclear blebs (double arrowhead). (**D**) Nuclear condensation (arrow) and extremely large lysosomal bodies (arrowhead). Note the swollen mitochondria (double arrowheads).

**Figure 9:**
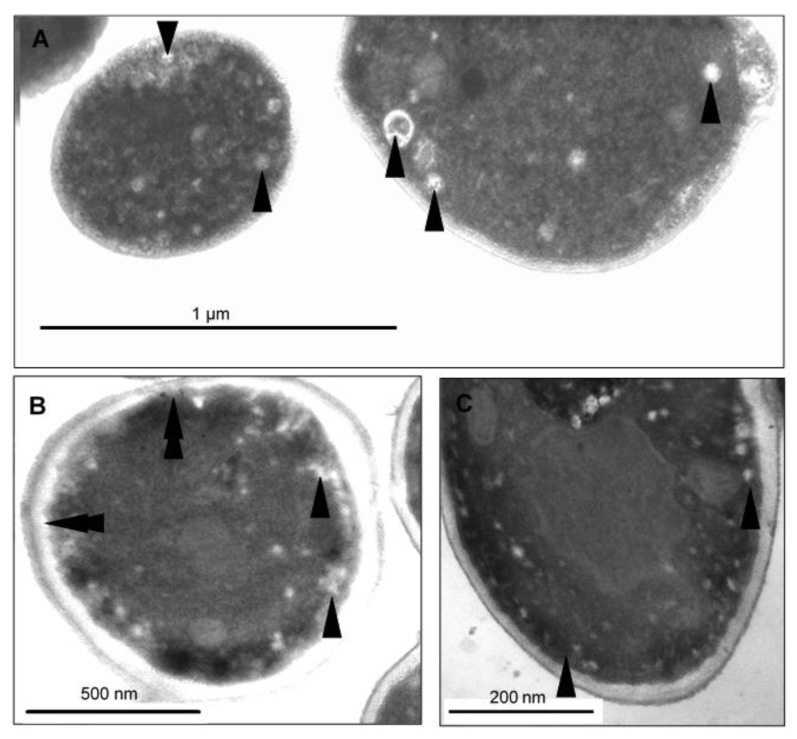
TEM micrographs of *C. glabrata* SZMC 1374 treated with a sub-lethal concentration of CAPE. (**A**–**C**) Mainly smaller necrotic signs were detected. Arrowheads denote small, mainly peripheral vacuoles typical of all samples. (**B**) Cell wall disintegration was also observed (double arrowhead).

To visualize the changes in intracellular morphology of the cells after CAPE treatment, transmission electron microscopy imaging was performed on *Candida* cells exposed to sub-lethal concentrations of CAPE. The TEM micrographs of *C. tropicalis* SZMC 1366, *C. albicans* SZMC 1423, and *C. parapsilosis* SZMC 8007 (Figure 6, Figure 7 and Figure 8, respectively) mainly revealed typical hallmarks of apoptosis, including nuclear chromatin margination, nuclear blebs, condensation in the nucleus, vacuolization, plasma membrane detachment, enlarged lysosomes, cytoplasm fragmentation, cell wall distortion, and whole-cell shrinkage. However, very few cells displayed signs of necrosis, such as membrane disintegration and loss of cytoplasm density, whereas the TEM micrographs of *C. glabrata* SZMC 1374 (Figure 9) mainly revealed smaller necrotic signs.

## 3. Discussion

This study focused on the antifungal and anti-biofilm effects of CAPE, which is one of the main biologically active components of propolis, on different *Candida* species including *C. albicans* and non-albicans *Candida* species. Moreover, we also investigated some of the mechanisms that might be involved in CAPE-induced cell death.

The *Candida* spp are still considered the most important opportunistic fungal pathogens that cause fungal infections worldwide. They are among the fourth to sixth most common nosocomial bloodstream isolates, according to estimates. Although *C. albicans* was the most frequent species isolated during candidemia, a greater role of non-albicans *Candida* spp has been observed in recent years [6,27]. Moreover, the extensive use of azole antifungals has resulted in the development of multidrug resistance in many *Candida* strains [28,29]. This resistance could be attributed to a change in drug intracellular accumulation, a change in membrane sterol composition, a change in efflux pump performance, or a change in ERG11 (the gene that is responsible for the production of the lanosterol-14-demethylase enzyme, the target of these medications) [6].

The biological activities of CAPE have been widely studied. However, limited number of studies were found in the literature concerning the antifungal activity. This study aimed to add some information about the activity of CAPE against *C. albicans* and non-albicans *Candida* species. Our findings showed that CAPE has high abilities to inhibit planktonic growth and biofilm formation as well as an ability to partially eradicate the mature biofilms of the different strains of *Candida*. Those results are in agreement with the results obtained by De Barros and coworkers, where the ability of CAPE to inhibit the growth of both fluconazole-sensitive and fluconazole-resistant strains of *C. albicans* has been reported [23]. Another study performed by Possamai Rossatto and coworkers showed similar results, where CAPE was also able to inhibit the growth of *C. auris* with MIC values ranging from 1 to 64 µg/mL. Furthermore, CAPE was able to inhibit the biofilm formation and phospholipase production of *C. auris* [30]. Our findings also showed the ability of CAPE to enter the cells of *Candida* spp rapidly. Such results were also observed by Cigut and coworkers in the yeast *Saccharomyces cerevisiae*. They found that, out of the four examined compounds (caffeic acid, p-coumaric acid, ferulic acid, and CAPE), CAPE was the only one that was able to enter the *S. cerevisiae* cells [31].

Phenolic compounds, including CAPE, are effective inhibitors of iron absorption [25]. According to Sun and coworkers, the antifungal mechanism of CAPE may include intracellular iron starvation due to its ability to form insoluble complexes with iron ions, which leads to the prevention of iron absorption by cells [25]. Using *C. albicans*-infected nematodes, Breger and coworkers showed that CAPE was able to inhibit the in vivo filamentation of *C. albicans*, leading to prolonged survival of infected nematodes [32]. Other studies attributed the antifungal activity of CAPE to its action on RNA, DNA, and cellular proteins, which are probable targets of this compound [13]. On the other hand, Su and coworkers suggested that the cytotoxicity of CAPE could be related to its apoptotic effect on the cells [33]. Marin and coworkers found that CAPE was able to induce the genes that are responsible for apoptosis and oxidative stress response. They reported that some polyphenolic compounds may have pro-oxidant activity, which can induce oxidative stress in the cells through the production of high levels of reactive oxygen species or inhibition of the system antioxidants [34]. In our study, we found that CAPE can induce apoptosis in five *Candida* strains, which are *C. albicans* ATCC 44829, *C. albicans* SZMC 1423, *C. albicans* SZMC 1424, *C. parapsilosis* SZMC 8007, *C. tropicalis* SZMC 1366, and *C. tropicalis* SZMC 1512. Surprisingly, *C. parapsilosis* SZMC 8007, *C. glabrata* SZMC 1374, and *C. glabrata* SZMC 1378 did not exhibit apoptotic cell death, which indicates that different species of *Candida* and, in one case, different strains of the same species have different cell death responses to CAPE. Moreover, the TEM images of CAPE-treated *Candida* cells showed the typical hallmarks of apoptosis in most *Candida* species. Notably, the apoptotic hallmarks were almost the same in different *Candida* species. Similar apoptotic hallmarks have been reported by several previous studies on *C. albicans*. De Nollin and Borgers (1975) reported the alterations of the surface micromorphology in *C. albicans* after treatment with miconazole. Shrinkage of protoplasm, abnormal cell and nuclear morphology, and vacuolization were also observed in plantaricin peptide-treated cells of *C. albicans* [35]. Distortion of the cell walls and membranes, which caused alterations of the surface micromorphology, could be explained due to a change in the permeability of the cell membrane, which could cause an osmotic imbalance, leading to alterations and indentations of the cell wall in collapsed cells [36].

Furthermore, we investigated the mechanisms involved in CAPE-induced apoptosis in *Candida* spp. Application of the broad-range pan-caspase inhibitor Z-VAD-FMK significantly reduced CAPE-induced apoptosis in *C. albicans* ATCC 44829, *C. albicans* SZMC 1424, *C. tropicalis* SZMC 1366, and *C. tropicalis* SZMC 1512. Such results suggest that this compound induced yeast caspase (Yca1p)-dependent apoptosis in these strains. Since CAPE can increase the permeability of the plasma membrane to ions [20], it can cause depolarization in mitochondria. This could lead to the release of cytochrome c and other proapoptotic factors into the cytosol, which in turn leads to the activation of yeast metacaspase Yca1p, resulting in the activation of caspase cascade inducing apoptosis [37]. However, the pre-incubation of CAPE-treated *C. albicans* SZMC 1423 and *C. parapsilosis* SZMC 8007 with the pan-caspase inhibitor Z-VAD-FMK did not affect their viability, which means that the CAPE-induced apoptosis in these strains was Yca1p-independent. This suggests that it could be due to the release of the apoptosis-inducing factor Aif1p from the mitochondria triggered by CAPE. These results support the fact that CAPE not only has species- and strain-dependent cell death responses in *Candida* but also could induce apoptotic cell death through different mechanisms.

## 4. Materials and Methods

### 4.1. Materials

For our experiments, CAPE (Sigma-Aldrich, Buchs, Switzerland); sodium dodecyl sulfate; crystal violet; peptone; yeast extract (Merck, Germany); agar-agar (Fluka, Buchs, Switzerland); a modified version of RPMI 1640 medium (containing dextrose 1.8% (*w*/*v*), MOPS 3.4% (*w*/*v*), and adenine 0.002% (*w*/*v*)) (Sigma-Aldrich, St. Louis, MI, USA); potassium dihydrogen phosphate; disodium hydrogen phosphate (Reanal, Budapest, Hungary); dimethyl sulfoxide; ethanol (VWR Chemicals, Paris, France); sodium chloride (VWR Chemicals, Debrecen, Hungary); glucose (VWR Chemicals, Leuven, Belgium); adenine; calcium chloride; magnesium chloride; potassium chloride (Scharlau Chemie S.A, Sentmenat, Spain); Z-VAD-FMK (Biovision, Milpitas, CA, USA); glutardialdehyde solution; osmium tetroxide; propylene oxide; Durcupan (R) ACM components A/M, B, C, and D (Sigma-Aldrich, Darmstadt, Germany); 0.22 µm vacuum filters (Merck Millipore, Guyancourt, France); sterile 96-well microtiter plates for susceptibility testing (Costar^®^, Phoenix, AZ, USA) and for biofilm assays (Sarstedt AG & Co. KG, Nümbrecht, Germany, Catalog number: 83.3924.500); CF^®^488A Annexin V and PI apoptosis Kit (Biotium, Fremont, CA, USA); and methanol (Chemolab Ltd., Budapest, Hungary) were used. All of the chemicals used in the experiments were of analytical or spectroscopic grade.

### 4.2. Instruments Used in the Experiments

A Thermo Scientific Heraeus B12 incubator (Thermo Fisher Scientific, Waltham, MA, USA), Sanyo orbital incubator (Sanyo, Japan), Sanyo autoclave (Sanyo, Japan), Hitachi U-2910 UV/Vis spectrophotometer (Hitachi, Japan), WTW pH meter (inoLab, Germany), Multiskan EX plate reader (Thermo Fisher Scientific Inc., Vantaa, Finland), benchtop centrifuge (Hettich, Buford, GA, USA), Ultramicrotome Reichert Jung Ultracut E (LabX, Midland, ON, Canada), JEOL-1200EX Transmission electron microscope (TEM), and Attune NxT flow cytometer (Thermo Fisher Scientific Inc., Massachusetts, USA) were used throughout the experiments. 

A Thermo Scientific Heraeus B12 incubator (Thermo Fisher Scientific, Waltham, MA, USA), Sanyo orbital incubator (Sanyo, Japan), Sanyo autoclave (Sanyo, Japan), Hitachi U-2910 UV/Vis spectrophotometer (Hitachi, Japan), WTW pH meter (inoLab, Germany), Multiskan EX plate reader (Thermo Fisher Scientific Inc., Vantaa, Finland), benchtop centrifuge (Hettich, USA), Ultramicrotome Reichert Jung Ultracut E (LabX, Canada), JEOL-1200EX Transmission electron microscope (TEM), and Attune NxT flow cytometer (Thermo Fisher Scientific Inc., Massachusetts, USA) were used throughout the experiments. 

### 4.3. Test Microorganisms, Culture Media, and Growth Conditions

Four species of *Candida* were used: *C. albicans*, *C. tropicalis*, *C. glabrata*, and *C. parapsilosis*. Nine strains were included, one of which was the American Type Culture Collection (ATCC) strain, and the others were *Candida* isolates obtained from Szeged Microbial Collection (SZMC), University of Szeged, Hungary (Table 2). All strains were maintained at the Department of General and Environmental Microbiology, Institute of Biology, University of Pécs, Hungary. All strains were grown in yeast extract peptone dextrose (YPD) broth (yeast extract 1%, peptone 2%, and glucose 2% in distilled water, pH 6.8) or on YPD plates.

### 4.4. Preparation of Stock Solution of CAPE

The stock solution was freshly prepared by dissolving CAPE in ethanol at a concentration of 10 mg/mL. The stock solution was kept in the freezer at −20 °C.

### 4.5. Antifungal Susceptibility Testing

The broth microdilution method was performed to determine the minimal inhibitory concentration (MIC_80_) according to the protocol of the National Committee for Clinical Laboratory Standards Institute with some modifications [38]. Briefly, a standardized initial inoculum (10^6^ cells/mL) was applied in all experiments. The experiments were performed in sterile, flat-bottom 96-well microplates. To obtain final CAPE concentrations ranging from 400 to 3.125 µg/mL, equal volumes (100 µL) of cell suspension and CAPE-containing YPD medium were added into the wells. Negative controls (media and cell suspension without CAPE) and blanks (media with CAPE) were included in each experiment. The concentration of the solvent was constantly fixed at 1%. The plates were incubated at 35 °C, and the absorbance was measured after 48 h at 600 nm using a Multiskan EX plate reader. The MIC_80_ of CAPE was determined as the lowest concentration that causes an 80% reduction in the growth when compared with that of the negative control.

### 4.6. Biofilm-Forming Ability Assay

Biofilm formation assay was performed using the crystal violet staining method as described previously [39]. To inoculate the test microplates, a stationary-phase yeast culture was prepared using an inoculum size equivalent to 0.5 McFarland standard. The culture was shaken thoroughly and then diluted at 1:100 using RPMI-1640 medium. A series of two-fold dilutions were prepared from the stock solution of CAPE. In the test microplates, equal volumes (100 µL) of each dilution were added to identical volumes (100 µL) of the diluted cell suspensions to obtain final concentrations from 100 to 1.562 µg/mL CAPE in the wells. In each experiment, the negative controls and blanks were included. The concentration of the solvent was constantly fixed at 1%. The microplates were kept in an incubator at 35 °C for 48 h; afterward, the liquid part was discarded and the remaining biofilms were repeatedly washed with phosphate-buffered saline (PBS) (pH 7.4). Formalin in PBS 2% (*v*/*v*) was used to fix the biofilms; then, the crystal violet 0.13% (*w*/*v*) staining was applied for 20 min at room temperature. The excess crystal violet stain was discarded, and the wells were washed thoroughly and repeatedly with PBS buffer. The estimation of biofilm mass was conducted by adding sodium dodecyl sulfate (SDS) in ethanol (1% *w*/*v*) solution to each well to extract the stain overnight, and the absorbance of the solution was measured at 600 nm using a Multiskan EX plate reader. The minimum biofilm inhibitory concentration (MBIC) was defined as the lowest concentration of CAPE that was able to inhibit 90% of the biofilm-forming ability.

### 4.7. Biofilm Eradication Assay

The effect of CAPE on mature biofilms was verified as described by Nostro and coworkers [40]. Briefly, for the inoculation of the assay microplates, stationary-phase yeast cultures were prepared using an inoculum size equivalent to 0.5 McFarland standard and thereafter diluted 1:100 using RPMI-1640 medium. Microplates containing diluted cell suspension were kept in an incubator at 35 °C for 48 h. After the biofilm maturation, CAPE treatment was applied. Accordingly, the original RPMI culture was discarded and replaced with a CAPE-containing RPMI medium with concentrations ranging from 100 to 1.562 µg/mL. Negative controls and blanks were included in each experiment. The concentration of the solvent was constantly fixed at 1%. After 48 h of incubation at 35 °C, the liquid part of the media was discarded, and the remaining biofilms were washed, fixed, stained, and estimated as mentioned in the previous section (Section 4.6).

### 4.8. Biosorption of CAPE by Candida Cells

To determine the cellular biosorption of CAPE, YPD broth cultures of *Candida* strains were grown overnight at 35 °C and 150 rpm in an orbital shaker. The number of yeast cells was adjusted to 10^7^ cells/mL in each case, and the cultures were treated with 100 µg/mL CAPE and incubated at 35°C with shaking at 150 rpm. The concentration of the solvent was constantly fixed at 1%. Samples were taken at the time points 0, 5, 10, 15, 20, 30, 60, and 120 min after admission and centrifuged (5000 rpm, 5 min), and the absorbance of the cell-free supernatants was measured at 330 nm (absorption maximum of CAPE) using a Hitachi U-2910 UV/Vis spectrophotometer. A calibration curve of two-fold serial dilutions of CAPE from 100 to 0.781 µg/mL was constructed and used to evaluate the biosorption levels of *Candida* cells [41].

### 4.9. Cell Death Examination Assay

The YPD broth media were inoculated with *Candida* cells (10^6^ cell/mL) from fresh YPD plate cultures and incubated at 35 °C with shaking at 150 rpm until reaching the mid-exponential phase depending on their growth curves. Media containing sub-lethal concentrations (MIC_80_) of CAPE were inoculated with 2.5 × 10^6^ cells/mL of the mid-exponential phase cultures of different *Candida* species and strains and incubated at 35 °C with shaking for 3 h. Untreated cell samples were included as negative controls in each experiment. The concentration of the solvent was constantly fixed at 1% in all experiments. After the incubation period, cells were harvested and washed with PBS. The CF^®^488A Annexin V and PI apoptosis Kit was used according to the manufacturer’s instructions to identify apoptosis and necrosis. In brief, *Candida* cells were re-suspended in 1X annexin V binding buffer at a concentration of 5 × 10^6^ cells/mL. To 100 µL of this solution, 5 µL of CF^®^488A Annexin V and 2 µL of PI working solution were added. The tubes were gently vortexed and incubated for 20 minutes at room temperature in the dark. After incubation, 400 µL of 1X annexin V binding buffer was added to each tube and analyzed using an Attune NxT flow cytometer. Annexin V is responsible for the detection of phosphatidylserine translocation from the inner to outer leaflets of the plasma membrane, whereas PI is a membrane-impermeant DNA-binding dye that is usually used to selectively stain dead cells in a cell population. PI is excluded by living cells and early apoptotic cells but stains necrotic and late apoptotic cells with compromised membrane integrity.

### 4.10. Caspase Inhibitor Assay

Caspase inhibitor assay was performed as described by Yue and coworkers [42] with some modifications. Briefly, cells were divided into two groups. The first group was pretreated for 1 h at 35 °C with the broad-spectrum caspase inhibitor Z-VAD-FMK (final concentration 77 µM) before incubation with CAPE. The second group was used as a control (not treated with the caspase inhibitor). For microplate assays, the cells were harvested by centrifugation, washed twice with PBS, and then re-suspended in YPD broth. The cell density was adjusted to 2 × 10^6^ cell/mL. Equal volumes (100 µL) of cell suspension and CAPE-containing YPD medium were dispensed into the wells to obtain the final CAPE concentration equal to the MIC_80_ of each strain. Negative controls (media and cell suspension without CAPE) and blanks (media with CAPE) were included in each experiment. The concentration of the solvent was constantly fixed at 1%. The plates were incubated at 35 °C, and the absorbance was measured after 48 h at 600 nm using a Multiskan EX plate reader.

### 4.11. Ultrastructural Examination of Candida Species by TEM

Media containing sub-lethal concentrations of CAPE were inoculated with 2.5 × 10^6^ cells/mL of the mid-exponential phase cultures of different species of *Candida* and incubated at 35 °C with shaking for 3 h to induce apoptosis. After the incubation period, the cells were harvested by centrifugation (5000 rpm, 5 min). The pellets were immediately washed and re-suspended with modified PBS (a mixture of 50 mM K_2_HPO_4_ and KH_2_PO_4_ (pH 7.0), supplemented with 0.5 mM MgCl_2_) and incubated at room temperature for 15 min to achieve equilibrium. Then, the samples were fixed overnight in 2.5% glutaraldehyde fixative buffered with modified PBS. The samples were then washed 4 times with modified PBS, and after short centrifugal sedimentation (1000 rpm, 2 min) preparation continued with a 2 % osmium tetroxide post-fixation on ice for 2 h. The cells were then washed twice with distilled water for 15 min and stained ‘en bloc’ in 1% aqueous uranyl acetate for 30 min. After two further washing steps with distilled water and short sedimentation, the cells were dehydrated in 70, 96, and 100% ethanol for 15 min each, subsequently. The cells were treated with propylene oxide twice for 10 min each time and then infiltrated for 1 h in a propylene oxide/Durcupan epoxy resin mixture (1:1) at room temperature. After 1 h, the cells were transferred to fresh epoxy resin drops for another 1 h. The resin was then changed, and the samples were left overnight in the fresh resin drops at room temperature. On the next day, the resin was changed twice while incubating at 40 °C for 2 h, subsequently. Finally, the samples were encapsulated in fresh epoxy resin and left at 56 °C for a two-day-long polymerization. Serial ultrathin sections were cut with Reichert Ultramicrotome, collected onto 300 mesh Nickel grids, counterstained on drops of uranyl acetate and Reynolds solution of lead citrate, washed thoroughly in sterile distilled water, and examined with a JEOL-1200 EX TEM at 80 KeV [43].

### 4.12. Statistical Analysis

All assays were carried out in triplicate, and data were expressed as mean ± standard deviation (SD). For data processing and visualization of the results, Microsoft Office Excel 2016 was used. Data were statistically analyzed through two-sample t-tests using Past3.21 software (University in Oslo, Oslo, Norway).

## 5. Conclusions

CAPE could be considered a promising natural antifungal agent. It has a concentration- and strain-dependent effect on the viability and biofilm-forming ability of the different *Candida* species. Moreover, it has a partial ability to eradicate the mature biofilms of biofilm-forming strains of *Candida*. In most *Candida* species and strains, the antifungal mechanism involves the induction of apoptotic cell death in treated cells. However, in other *Candida* species and strains, no apoptotic cell death was observed. This information suggests that CAPE may have a species- and strain-dependent cell death response in *Candida*. 

## Figures and Tables

**Table 1:** MIC_80_ values of CAPE against the different *Candida* strains.

Strain	MIC_80_ (µg/mL)
*C. albicans* ATCC 44829	50
*C. albicans* SZMC 1423	100
*C. albicans* SZMC 1424	50
*C. glabrata* SZMC 1374	12.5
*C. glabrata* SZMC 1378	12.5
*C. parapsilosis* SZMC 8007	25
*C. parapsilosis* SZMC 8008	12.5
*C. tropicalis* SZMC 1366	50
*C. tropicalis* SZMC 1512	50

**Table 2:** *Candida* species and strains used in the study.

Species	Collection Code	Origin	Biofilm-Forming Ability
*C. albicans*	ATCC 44829	auxotrophic mutant isolated after N-methyl N’-nitro-N-nitrosoguanidine treatment of a wild-type strain of *C. albicans*.	Non-biofilm forming
*C. albicans*	SZMC 1423	clinical sample/Debrecen, Hungary	Non-biofilm forming
*C. albicans*	SZMC 1424	clinical sample/Debrecen, Hungary	High-biofilm forming
*C. tropicalis*	SZMC 1366	hemoculture/Debrecen, Hungary	High-biofilm forming
*C. tropicalis*	SZMC 1512	-/Pécs, Hungary	Non-biofilm forming
*C. glabrata*	SZMC 1374	clinical sample/Debrecen, Hungary	High-biofilm forming
*C. glabrata*	SZMC 1378	clinical sample/Debrecen, Hungary	Non-biofilm forming
*C. parapsilosis*	SZMC 8007	clinical sample/Szeged, Hungary	High-biofilm forming
*C. parapsilosis*	SZMC 8008	unknown	Non-biofilm forming

## Data Availability

Not applicable.

## Supplementary Materials

The following are available online at https://www.mdpi.com/article/10.3390/antibiotics10111359/s1, Figure S1: Representative flow cytometry scatter plot showing apoptosis of *C. albicans* ATCC 44829 treated with 50 µg/mL of CAPE, Figure S2: Representative flow cytometry scatter plot showing apoptosis of *C. albicans* SZMC 1423 treated with 100 µg/mL of CAPE, Figure S3: Representative flow cytometry scatter plot showing apoptosis of *C. albicans* SZMC 1424 treated with 50 µg/mL of CAPE, Figure S4: Representative flow cytometry scatter plot showing apoptosis of *C. parapsilosis* SZMC 8007 treated with 25 µg/mL of CAPE, Figure S5: Representative flow cytometry scatter plot showing apoptosis of *C. parapsilosis* SZMC 8008 treated with 12.5 µg/mL of CAPE, Figure S6: Representative flow cytometry scatter plot showing apoptosis of *C. glabrata* SZMC 1374 treated with 12.5 µg/mL of CAPE, Figure S7: Representative flow cytometry scatter plot showing apoptosis of *C. glabrata* SZMC 1378 treated with 12.5 µg/mL of CAPE.

## Abbreviations

CAPE: Caffeic Acid Phenethyl Ester; MIC, Minimal Inhibitory Concentration; MBIC, Minimal Biofilm Inhibitory Concentration; PBS, Phosphate-Buffered Saline; PI, Propidium Iodide; YPD, Yeast extract Peptone Dextrose.
