# Supplementary material for: Antifungal and Anti-Biofilm Effects of Caffeic Acid Phenethyl Ester on Different Candida Species
Source: Antibiotics (Basel). 2021 Nov 7;10(11):1359. doi: 10.3390/antibiotics10111359 (PMC8614700; doi:10.3390/antibiotics10111359)
Supplement: Supplementary file 1 [file antibiotics-10-01359-s001.zip › Supplementary materials.pptx]

## Slide 1
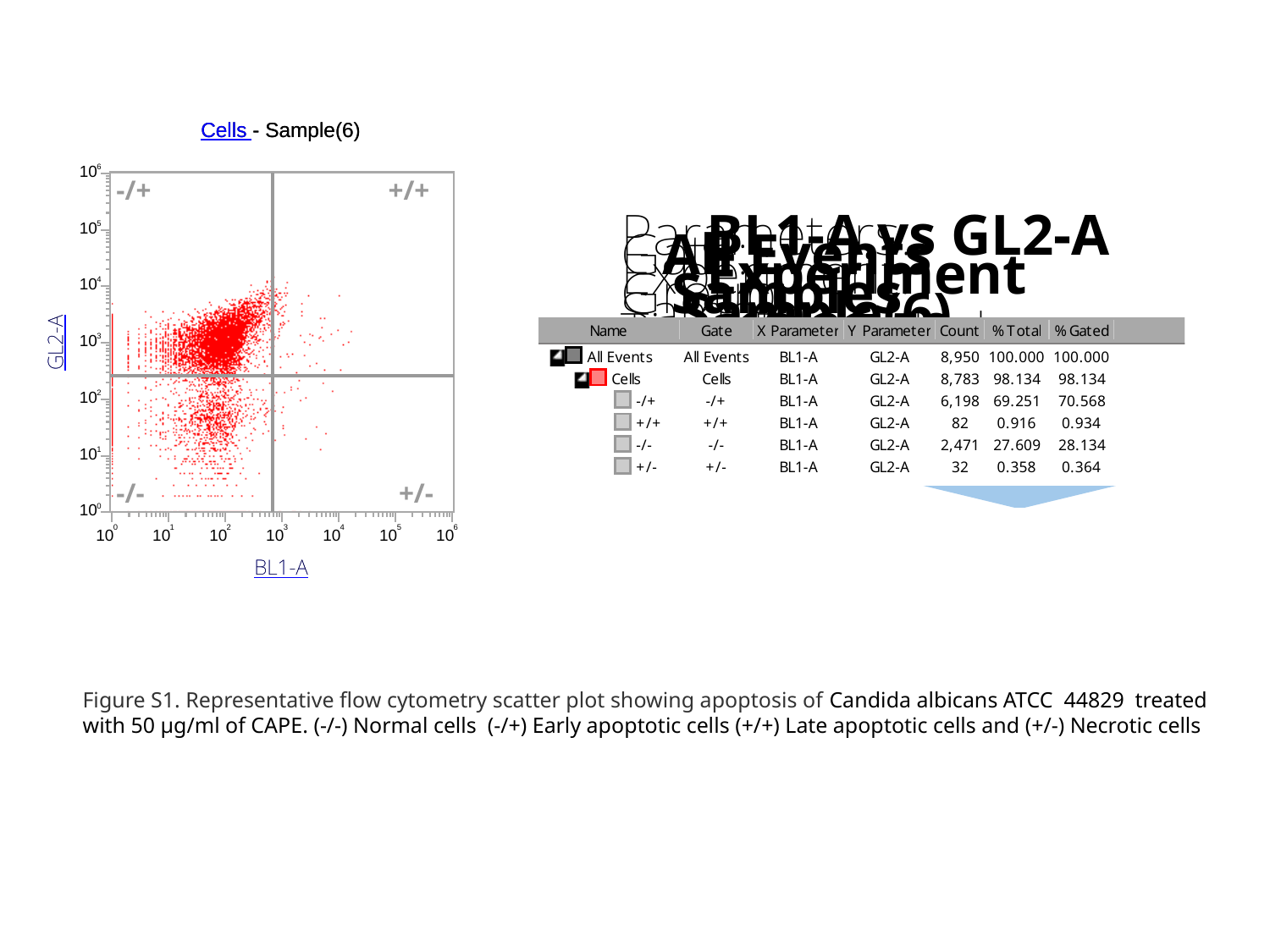

Figure S1. Representative flow cytometry scatter plot showing apoptosis of Candida albicans ATCC 44829 treated with 50 µg/ml of CAPE. (-/-) Normal cells (-/+) Early apoptotic cells (+/+) Late apoptotic cells and (+/-) Necrotic cells

## Slide 2
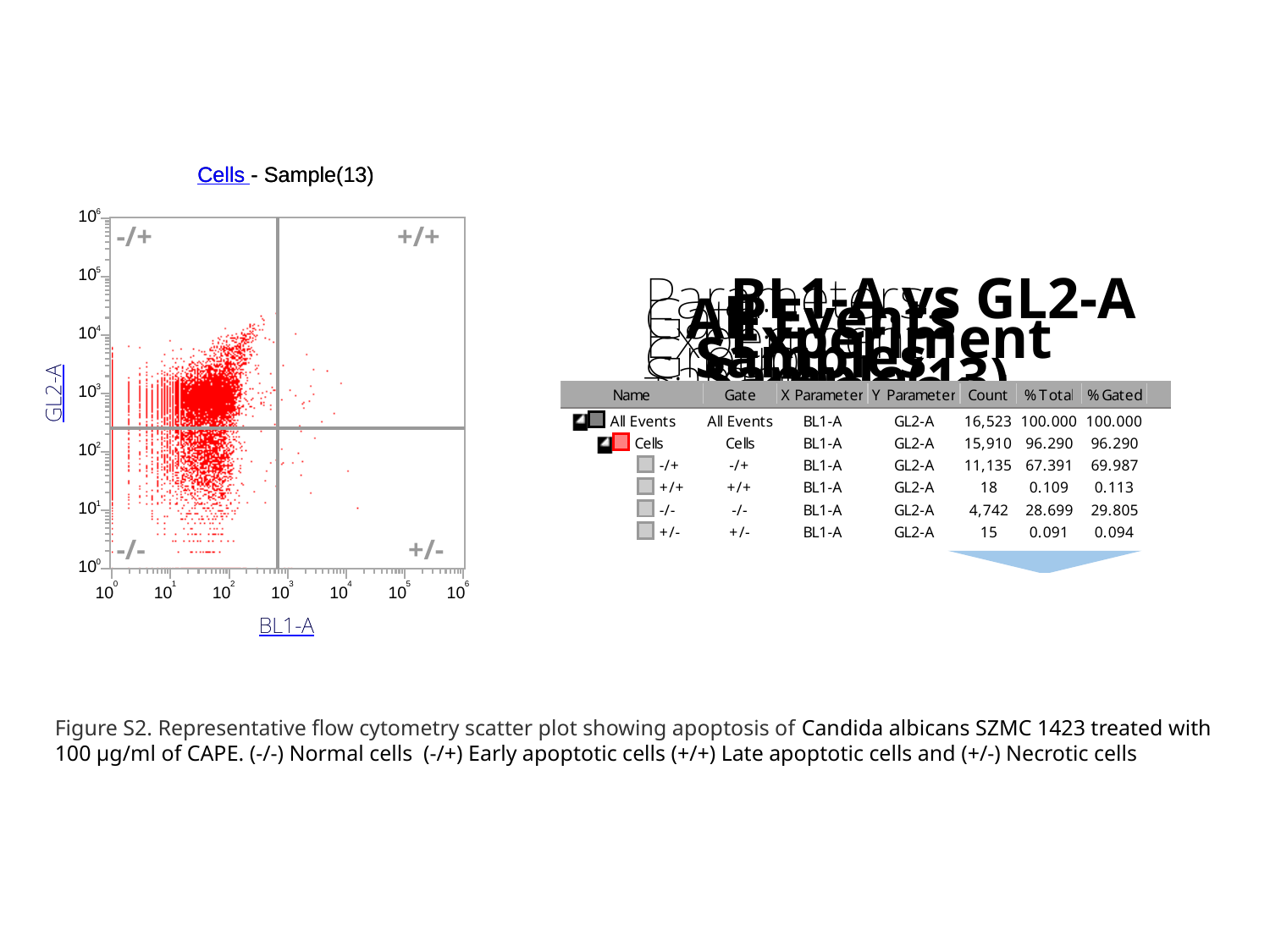

Figure S2. Representative flow cytometry scatter plot showing apoptosis of Candida albicans SZMC 1423 treated with 100 µg/ml of CAPE. (-/-) Normal cells (-/+) Early apoptotic cells (+/+) Late apoptotic cells and (+/-) Necrotic cells

## Slide 3
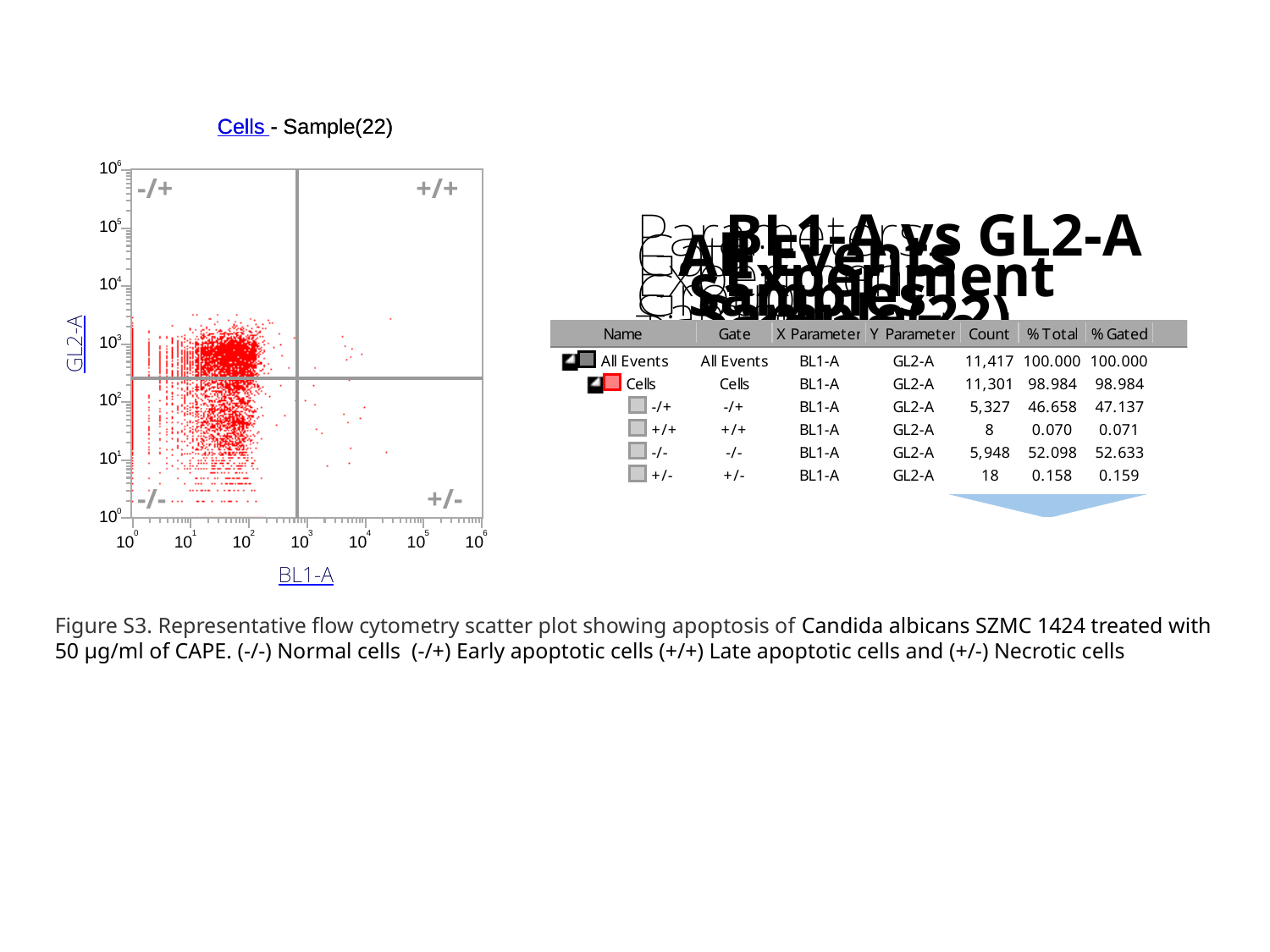

Figure S3. Representative flow cytometry scatter plot showing apoptosis of Candida albicans SZMC 1424 treated with 50 µg/ml of CAPE. (-/-) Normal cells (-/+) Early apoptotic cells (+/+) Late apoptotic cells and (+/-) Necrotic cells

## Slide 4
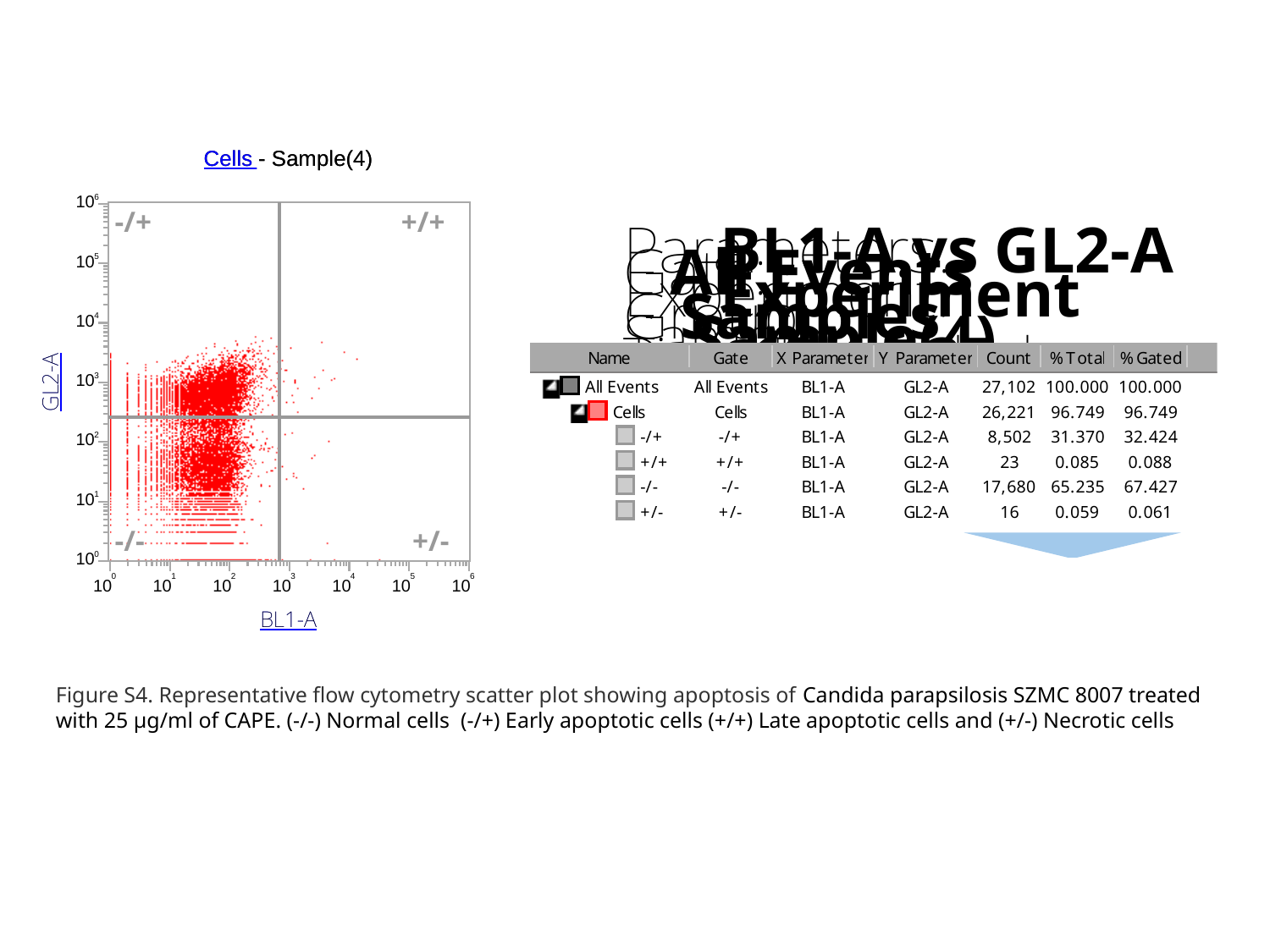

Figure S4. Representative flow cytometry scatter plot showing apoptosis of Candida parapsilosis SZMC 8007 treated with 25 µg/ml of CAPE. (-/-) Normal cells (-/+) Early apoptotic cells (+/+) Late apoptotic cells and (+/-) Necrotic cells

## Slide 5
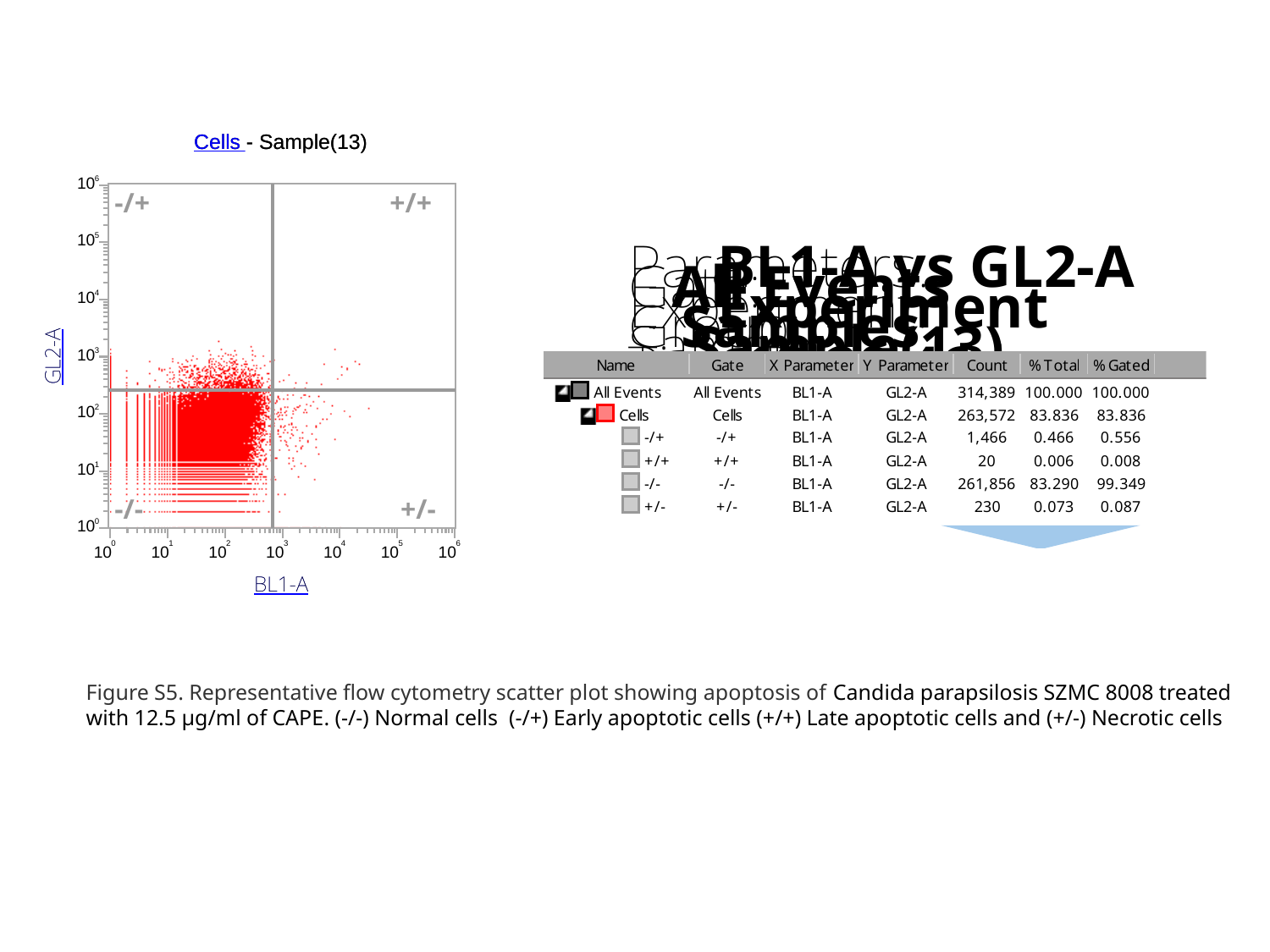

Figure S5. Representative flow cytometry scatter plot showing apoptosis of Candida parapsilosis SZMC 8008 treated with 12.5 µg/ml of CAPE. (-/-) Normal cells (-/+) Early apoptotic cells (+/+) Late apoptotic cells and (+/-) Necrotic cells

## Slide 6
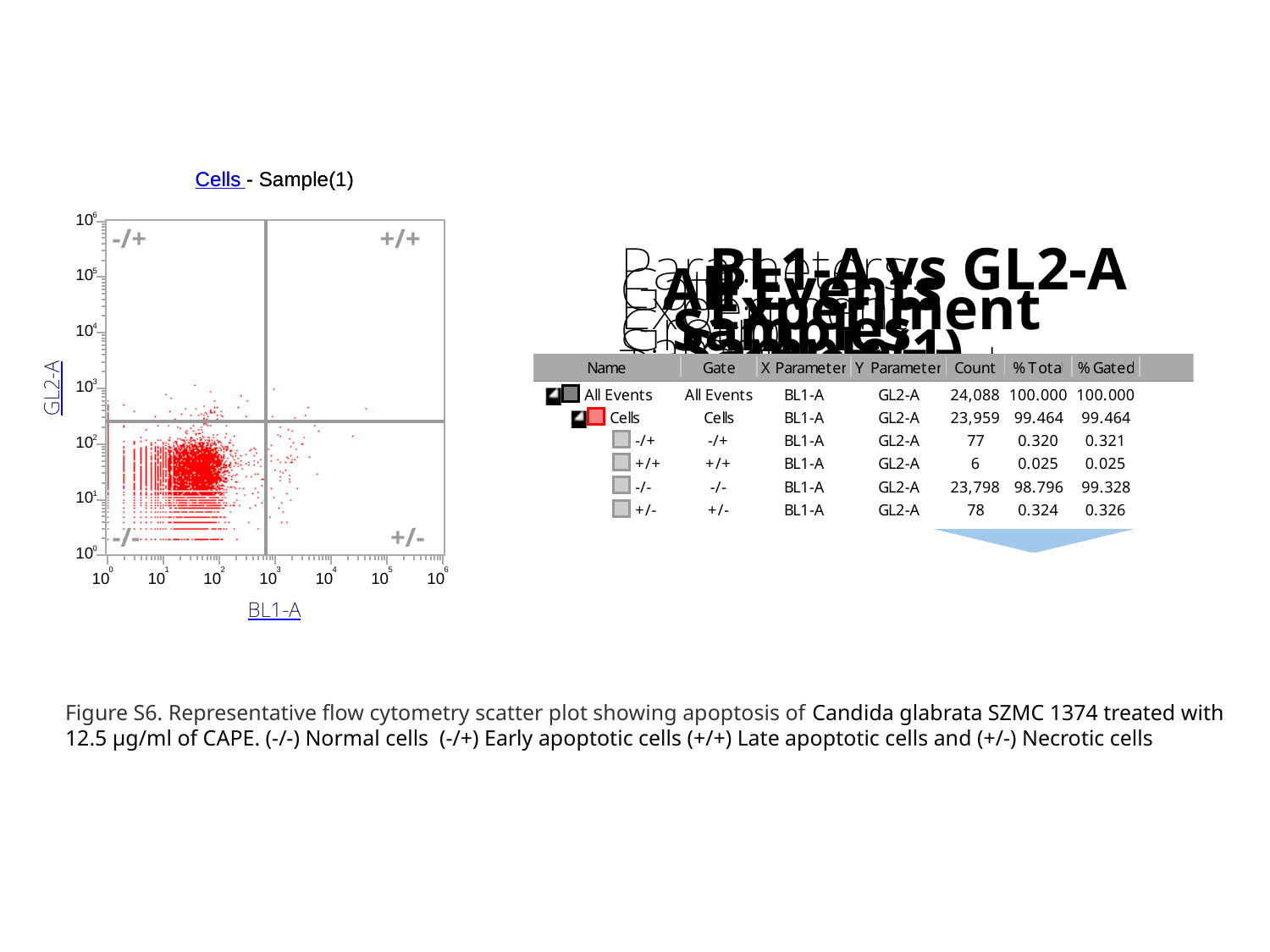

Figure S6. Representative flow cytometry scatter plot showing apoptosis of Candida glabrata SZMC 1374 treated with 12.5 µg/ml of CAPE. (-/-) Normal cells (-/+) Early apoptotic cells (+/+) Late apoptotic cells and (+/-) Necrotic cells

## Slide 7
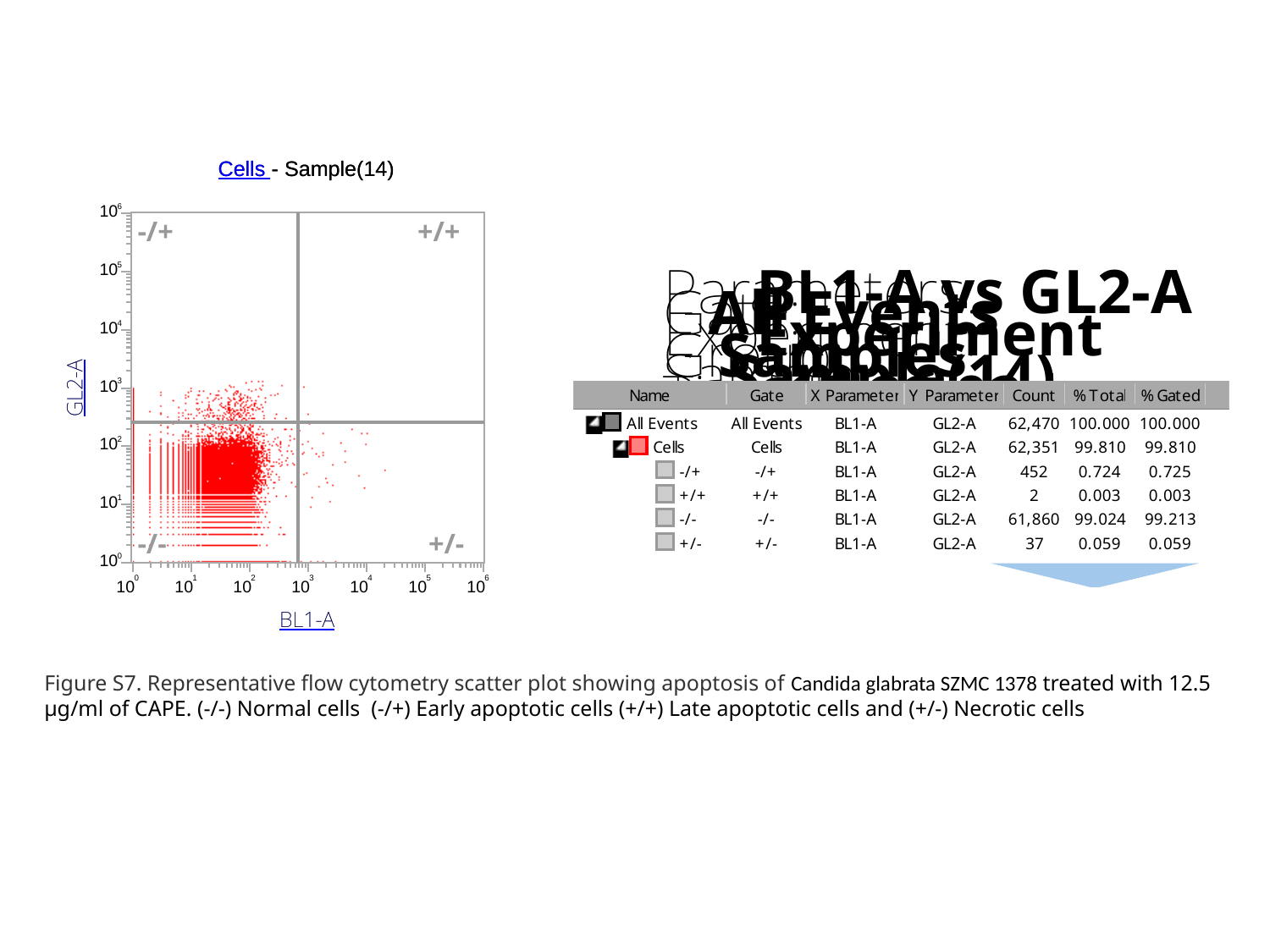

Figure S7. Representative flow cytometry scatter plot showing apoptosis of Candida glabrata SZMC 1378 treated with 12.5 µg/ml of CAPE. (-/-) Normal cells (-/+) Early apoptotic cells (+/+) Late apoptotic cells and (+/-) Necrotic cells
